# Fe@χ_3_-borophene as a promising catalyst for CO oxidation reaction: A first-principles study

**DOI:** 10.3389/fchem.2022.1008332

**Published:** 2022-09-13

**Authors:** Jian-Wei Han, Wei-Yue Bian, Yue-Yu Zhang, Meng Zhang

**Affiliations:** ^1^ School of Physics, East China University of Science and Technology, Shanghai, China; ^2^ Wenzhou Institute, University of Chinese Academy of Sciences, Wenzhou, Zhejiang, China

**Keywords:** CO oxidation reaction, borophene, first-principles study, catalytic activity, reaction mechanism

## Abstract

A novel single-atom catalyst of Fe adsorbed on χ_3_-borophene has been proposed as a potential catalyst for CO oxidation reaction (COOR). Quantitative pictures have been provided of both the stability of Fe@χ_3_-borophene and various kinetic reaction pathways using first-principles calculations. Strong adsorption energy of -3.19 eV and large diffusion potential of 3.51 eV indicates that Fe@χ_3_-borophene is highly stable. By exploring reaction mechanisms for COOR, both Eley-Ridel (E-R) and trimolecule E-R (TER) were identified as possible reaction paths. Low reaction barriers with 0.49 eV of E-R and 0.57 eV of TER suggest that Fe@χ_3_-borophene is a very promising catalyst for COOR. Charge transfer between the χ_3_-borophene and CO, O_2_ and CO_2_ gas molecules plays a key role in lowering the energy barrier during the reactions. Our results propose that Fe@χ_3_-borophene can be a good candidate of single-atom catalyst for COOR with both high stability and catalytic activity.

## Introduction

The CO oxidation reaction (COOR) is a simple yet fundamental reaction to reveal the intrinsic mechanism of multiphase catalytic conversion reactions and to test the reactivity of new catalysts (Alavi et al., 1998). The conversion of CO to non-toxic gases at room temperature has attracted considerable attention in recent years due to the seriously growing environmental problems and energy shortages (Ernst and Zibrak, 1998; Prockop and Chichkova, 2007). In the past few years, many studies have been focusing on the mechanism of COOR using noble metal nanoparticles and alloys as catalysts such as Pd (Kalita and Deka, 2009), Ag (Kim et al., 2010), Pt (Zhang and Hu, 2001), Au (Lopez and Nørskov, 2002) and others. However, due to the high cost and scarcity of these metals, they have never been commercialized. Therefore, it is crucial to find suitable catalytic methods and catalysts for COOR to improve the catalytic effect and reduce the cost.

A new class of heterogeneous catalysis emerges in the past decade by supporting metal nanoparticles on a surface. Among these systems, single-atom catalysts (SACs) on two-dimensional substrates has been a popular research topic since the single-atom catalyst Pt_1_/FeO_x_ was first reported to be synthesized in 2009 by Zhang et al. (Qiao et al., 2011). The advantages of SACs include clear active sites, high utilization of metals, high-efficiency in catalytic performance, wide applicability and so on (Yang et al., 2013; Liu et al., 2018; Wang et al., 2018). A fundamental understanding of the reaction pathways on SACs can not only further guide the experimentalist to find more efficient catalysts but also provide valuable theoretical research ideas for COOR.

Due to the outstanding stability and large surface area, graphene has become a promising candidate for anchoring SACs since its discovery in 2004 (Wang et al., 2012). Up to now, the COOR of the noble metal [Au (Lu et al., 2009), Cu (Song et al., 2011), Pt (Tang et al., 2013)], and other transition metal atoms (Pd (Jia et al., 2014), Mo (Tang et al., 2015)) adsorbed or doped on the surface of graphene has been studied successively. In these systems, the reaction path based on Langmuir–Hinshelwood (L-H) mechanism (Lu et al., 2009), Eley-Ridel (E-R) mechanism (Tang et al., 2017) and trimolecule E-R (TER) mechanism (Zhang et al., 2015) has been confirmed. Recently, an increasing amount of efforts has been devoted to finding catalysis for SACs based on other two-dimensional materials, such as graphyne (Wu et al., 2015), MoS_2_ (Du et al., 2015), and hexagonal boron nitride monolayer (h-BN) (Lin et al., 2013; Liu et al., 2014), or to modify graphene to change some of the carbon atoms to N and other atoms (Zhang et al., 2016; Liu et al., 2017), thereby changing the catalytic environment of single metal to achieve better catalytic performance.

As a neighbor of carbon, borophene has attracted increasing interest since it was synthesized recently on a silver substrate under ultrahigh-vacuum with four phases including 2-Pmmn, β_12_, χ_3_, and honeycomb phases (Tang and Ismail-Beigi, 2007; Yang et al., 2008; Mannix et al., 2015). Borophene has complex bonding mechanisms and multiple coordination capabilities (Wang et al., 2019). Particularly, the adsorption of metal atoms with electron-donating properties is expected to occur more likely on borophene than on graphene because of the electron deficient property of boron atoms (Liu et al., 2013). Due to the unique physical and chemical properties, borophene has pronounced potential applications in the service of SACs as the substrate. Based on density functional theory, nitrogen reduction reaction (Liu et al., 2019a; Zhu et al., 2019), hydrogen evolution reaction (Xu et al., 2020a), oxygen evolution/reduction reaction (Xu et al., 2021) and electrocatalytic CO_2_ reduction (Shen et al., 2018; Zhang et al., 2020) about SACs have been extensively studied on different phases of borophene, and promising catalytic results have been obtained, indicating that borophene has great research potential for applications in the field of SACs.

However, these previous studies on the application of borophene on SACs mainly focus on electrocatalysis. Besides, most of these works are about α-borophene and β-borophene phase which are considered as the thermodynamically most stable phase based on the density functional theory simulation (Wu et al., 2012). As far as we know, there are few studies about COOR of SACs on borophene, especially there is a lack of research on the catalytic performance of the other phases of borophene. Here, the χ_3_-borophene has been selected to investigate the SACs, which was successfully synthesized by Feng’s team in 2016 under ultra-high vacuum conditions (Feng et al., 2016). Fe cation has been reported with good catalytic activity for many reactions in the gas phase, while other cations such as Ti^+^, Zr^+^, V^+^, Nb^+^, Cr^+^ have no such activity (Staley, 1981). In spite of extensive investigations in SACs on two dimensional materials, the transition metal atoms with unfilled d electrons and robust magnetic properties are an interesting and challenging topic in catalysis. In this paper, the COOR of Fe atoms adsorbed on χ_3_-borophene are systematically investigated using density functional theory to explore the COOR effect. We believe that the results of this paper will provide new ideas for the catalytic properties of borophene and the study of COOR.

## Computational details

The first-principles calculations were carried out using the DMol^3^ package (Delley, 1990) embedded in Material Studio. Here we used χ_3_-borophene as substrate and a (2 × 4) supercell was built for simulation, which contains total 64 boron atoms. The Perdew–Burke–Ernzerhof (PBE) functional was used as the exchange-correlation functional under the generalized gradient approximation (GGA) (Perdew et al., 1996). The electronic eigenfunctions were expanded in terms of localized atomic orbital DNP (Delley, 1990) basis set, and the core treatment used was DFT semicore pseudopotentials (DSPPs) (Delley, 2002), which replaced the core electrons with individual effective potentials. The real-space cutoff radius was set to 4.7 Å to ensure high-quality results. In the process of geometric optimization, the energy convergence value was 1.0 × 10^−5^ Ha/atom, the maximum stress convergence value was 0.02 Ha/Å, the maximum displacement convergence value was 0.005 Å, and the threshold of self consistent-field (SCF) density convergence was 1.0 × 10^−6^ Ha. The K point of the Brillouin zone was selected as 2 × 2 × 1 under the Monkhorst-Pack method (Monkhorst and Pack, 1976). The K point set for PDOS calculation which is 4 × 4 × 1 here in the Fe@χ_3_-borophene system. The vacuum layer between χ_3_-borophene layers was set to 20 Å to ensure that there was no interaction between layers. Van der Waals interaction was described by DFT + D2 method throughout all calculations (Grimme, 2006). Mulliken’s net charge analysis has been applied to count charge transfer between the adsorbate atoms and the substrate (Mulliken, 1955). The transition states (TSs) were searched by the linear both synchronous transit (LST) and quadratic synchronous transit (QST) protocol. We first performed LST, and then repeated conjugate gradient minimization and QST maximization until the TS was found (Halgren and Lipscomb, 1977). To check the dynamic stability of the substrate, we ran the *ab-initio* molecular dynamics simulation using the NVT ensemble.

The adsorption energy E_ads_ of the adsorbent on the substrate is defined as:

$${\text{E}}_{ads}={\text{E}}_{adsorbate/substrate}-{\text{E}}_{adsorbate}-{\text{E}}_{substrate}$$ (1)

where E_adsorbate/substrate_ is the total energy of the system, E_adsorbate_ is the energy of the ground state of the adsorbate, and E_substrate_ is the energy of the substrate. In order to include the influence of temperature, the change in Gibbs free energy (ΔG) of reaction path is calculated according to

$$\Delta G=\Delta E+\Delta E_{ZPE}-\text{T}\Delta S$$ (2)

where ΔE and ΔE_ZPE_ are the difference between total energy at 0 K and the zero-point vibration energy, respectively. ΔS is the change of entropy, defined as ΔS = ΔS_trans_ + ΔS_rot_ + ΔS_vib_, where ΔS_trans_, ΔS_rot_, and ΔS_vib_ represent the contribution of translation, rotation, and vibration modes, respectively.

According to the transition state theory, the time required for each step of the reaction can be estimated by the Arrhenius law (Arrhenius, 1889):

$$\text{τ}=\frac{1}{{\text{νe}}^{\left(\frac{-E_{\text{a}}}{{\text{κ}}_{\text{B}}\text{T}}\right)}}$$ (3)

where E_a_ is the energy barrier of the reaction, T is the temperature (K), ν is the attempt frequency, and here the value we use is 10^12^ Hz, which is the same value used in similar systems (Xu et al., 2016; Xu et al., 2018), and $\kappa_{B}$ is the Boltzmann constant.

## Results and discussion

### Structural stability of Fe@χ_3_-borophene

**FIGURE 1:**
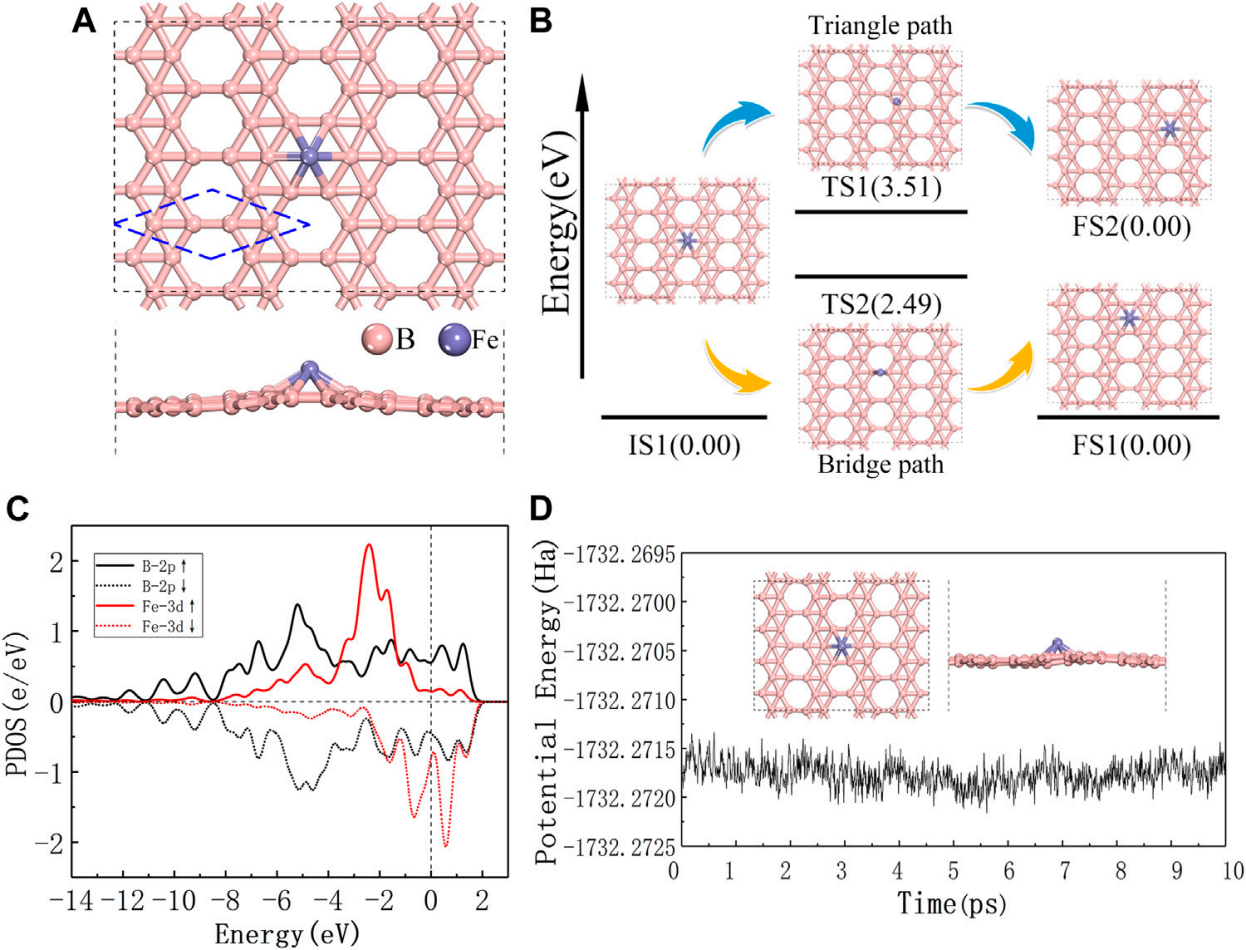
**(A)** Top and side views of the most stable configurations of single Fe atom adsorbed on χ_3_-borophene, the blue dashed line is the primitive cell of χ_3_-borophene. **(B)** The energy profile for Fe atom diffusion on χ_3_-borophene surface with the configurations involved. **(C)** The spin polarized partial density of states for Fe atom and the 6 B atoms around it, the vertical dashed line at 0 eV is the Fermi level. **(D)** *Ab-initio* molecular dynamics calculation of Fe@χ_3_-borophene at 300 K, the trajectory length is 10 ps, the inserted figure shows the structure of the system after 10 ps?

The thermodynamic stability of Fe@χ_3_-borophene is first studied before we further investigate the catalytic activity of this system for COOR. The atomic model of Fe@χ_3_-borophene include a single Fe atom adsorbed on χ_3_-borophene. The initial adsorption sites of a single Fe atom in the Fe@χ_3_-borophene are first placed at top-site, bridge-site or triangle-site and then optimized, as depicted in Figure 1A and Supplementary Figure S1. The result of geometric optimization shows that the heart-site is the most favorable site for Fe atom adsorption on χ_3_-borophene with an adsorption energy of -3.19 eV. The distance between the Fe atom at the heart-site and the adjacent B atom is 2.067 Å and the protruding height between the Fe atom and the χ_3_-borophene plane is 1.381 Å.

The diffusion behavior of Fe atoms on the χ_3_-borophene surface is studied by evaluating the energy barrier between one heart-site to a neighboring one. Two diffusion paths are identified as the Bridge path and the Triangle path and shown in Figure 1B. The calculated energy barriers of the Bridge path and Triangle path are 2.49 and 3.51 eV, respectively. Therefore, the Bridge path should be preferred as the diffusion path. Higher diffusion energy barrier is shown for Fe@χ_3_-borophene than those reported on graphene (Li et al., 2010), so that Fe atom is less favorable to migrate on χ_3_-borophene.

To study the self-aggregation of Fe on χ_3_-borophene, the structures and energies of nFe (n = 1–4) and Fe_n_ (n = 1–4) clusters adsorbed on the surface of both graphene and χ_3_-borophene (as shown in Supplementary Figures S2 and S3) are calculated for comparison. It can be seen from Supplementary Figures S2 and S3 that the total energy of isolated Fe atoms is significantly lower than that of Fe clusters on χ_3_-borophene, indicating that the structure of isolated Fe atoms anchored on χ_3_-borophene’s heart-site is favorited, while Fe tends to form clusters spontaneously on graphene. Thus, we could avoid the problem of self-aggregation of Fe atoms by choosing χ_3_-borophene as substrate.

The spin polarized partial density of states (PDOS) for Fe@χ_3_-borophene has been studied to further understand the interaction between Fe atom and χ_3_-borophene. PDOS in Figure 1C shows both the 3d-orbital of the guest Fe and the 2p-orbital of the host B atoms cross the Fermi level, showing the metallic characteristics of the Fe atom adsorbed on χ_3_-borophene system as demonstrated in the pure χ_3_-borophene (Feng et al., 2016). The superposition of one sharp peak originates from the d orbital of the adsorbed Fe atom and the p orbital of substrate B atoms span from -8.0 to 2.0 eV near the Fermi level. The strong hybridization shown in PDOS verifies the strong interaction between the guest Fe and host borophene and results in the high stability of the structure. We find that the shapes of the PDOS not only in d state of Fe atom but also in p state of borophene are quite different (see Figure 1C). Fe@χ_3_-borophene carries a large magnetic moment of 3.0 ${\text{μ}}_{\text{B}}$ , which is similar to the results obtained from the calculations with Fe adsorbed on different 2d borophene polymorphs (Alvarez-Quiceno et al., 2017; Liu et al., 2019b). Mulliken population analysis shows that the total magnetic moment of the clusters is mainly localized on the Fe atom. The χ_3_-borophene substrate provides a small contribution to the magnetic moments, as shown in Figure 1C. This can be ascribed to the internal charge transfer from the Fe to the B atoms. Due to the electron deficiency of χ_3_-borophene, the Fe atom is positively charged, donating about 1.34 e to the substrate. According to literatures (Lu et al., 2009), charge transfers not only contribute to the stability of the system, but also provide activity for subsequent catalytic reactions.

The stability of Fe@χ_3_-borophene under room temperature is confirmed by *ab-initio* molecular dynamics calculation (Figure 1D). Within the employed simulation time of 10 ps at 300 K, no structural change is detected, while the instantaneous values of the potential energy fluctuate slightly due to thermal fluctuations. Thus, the lowest energy structure of Fe@χ3-borophene is expected to be stable at room temperature.

### Adsorption properties of CO, O_2_ and CO_2_ on Fe@χ_3_-borophene

**FIGURE 2:**
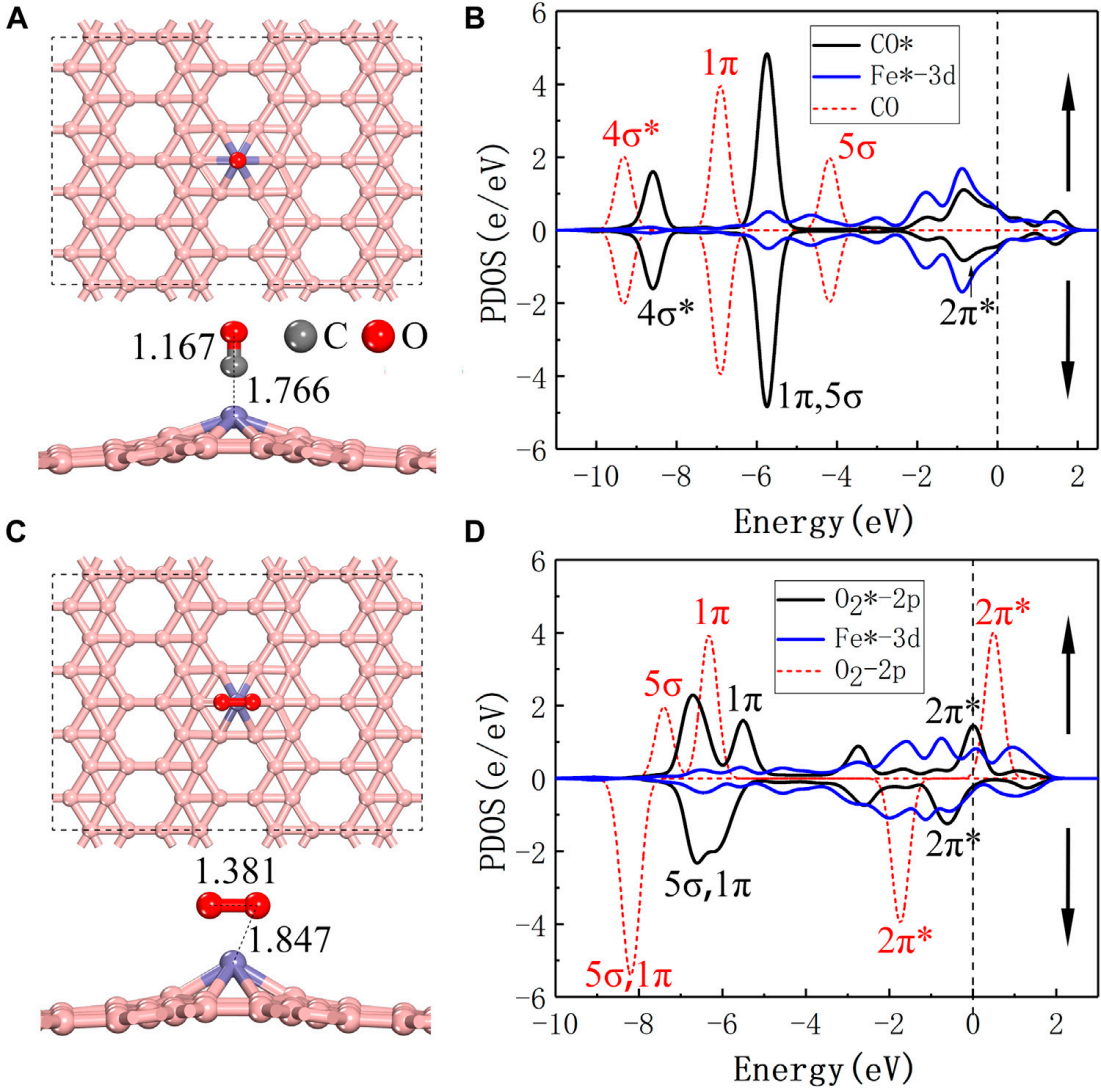
**(A,C)** are top and side views of the most stable configurations of CO/O_2_ adsorbed on Fe@χ_3_-borophene, respectively. **(B,D)** are the spin polarized partial density of states for the CO/O_2_ and Fe after adsorbed on Fe@χ_3_-borophene. The vertical dashed line at 0 eV is the Fermi level and the red dashed line is the PDOS for CO and O_2_ in the gas phase.

In order to understand the specific reaction path of COOR on the surface of Fe@χ_3_-borophene, it is important to further study the adsorption and co-adsorption properties of various gas molecules including CO, O_2_ and CO_2_ involved in COOR on Fe@χ_3_-borophene. The most stable adsorption configurations of various gas molecules absorbed on Fe@χ_3_-borophene are shown in Figure 2 and Supplementary Figure S4. Their adsorption energies, charge transfers and magnetic moments are listed in Table 1.

**TABLE 1:** The related parameters of gas molecules absorbed on Fe@χ_3_-borophene: adsorption energy (E_ads_), charge transfer between the Fe atom and the χ_3_-borophene (Δq_1_), charge transfer between gas molecules and Fe@χ_3_-borophene (Δq_2_), magnetic moment of the system.

*System*	*E*_ads_ (eV)	*Δq*_1_ (e)	*Δq*_2_ (e)	*Moment* ( ${\text{μ}}_{\text{B}}$ )
CO_2_	-0.30	1.37	-0.02	3.0
2CO_2_	-0.59	1.38	0.02	3.0
O_2_	-2.16	1.29	-0.56	2.0
CO	-2.44	0.94	-0.24	0.0
CO + O_2_	-3.15	1.14	-0.62	0.0
2CO	-4.29	0.95	-0.28	0.0
CO_3_	-5.24	1.31	-0.98	0.0

The most favorable adsorption sites for CO and O_2_ are shown in Figures 2A,C, respectively. Both the CO and O_2_ molecules adsorb on top of the Fe atom as they approach the Fe@χ_3_-borophene. For CO, the lowest energy adsorption model is that the CO molecule is located vertically above the Fe@χ_3_-borophene, where Fe, C, O atoms form a line and the C atom is bonded with the Fe atom. The bond length of CO* (1.167 Å) is slightly larger than that of gaseous CO (1.129 Å) and the distance between Fe and C is 1.766 Å (”*” represents the adsorption state). The most stable adsorption position for O_2_* is the horizontal configuration, where two O atoms are adsorbed on the Fe@χ_3_-borophene and form an inverted triangle with the Fe atom. The bond length of O_2_* increases significantly from 1.208 Å to 1.381 Å compared to the gas phase and the bond length of O and Fe is 1.847 Å. The lowest energy adsorption positions for other gas molecules or combinations of gas molecules including 2CO, CO + O_2_, CO_3_, CO_2_ and 2CO_2_ are drawn in Supplementary Figure S4.

The PDOS for CO-Fe@χ_3_-borophene is shown in Figure 2B. After the CO molecule is absorbed, the 3d spin-up and spin-down lines of Fe atom in the graph are almost identical in shape. A CO molecule adsorbed on the Fe@χ_3_-borophene is found to exhibit no magnetic moment, which means the magnetic moment of 3.0 ${\text{μ}}_{\text{B}}$ in the substrate is completely quenched. The 5σ orbital has similar energy levels to 1π orbital for the adsorbed CO molecule. The 2π* orbitals of CO are filled and have an obvious hybrid effect with the 3d orbitals of Fe atom, leading to a reduction in the energy of the system (Jiang et al., 2018; Jiang et al., 2020; Jiang et al., 2021). For O_2_-Fe@χ_3_-borophene, the empty 2π* anti-bond orbital is occupied, which results in a longer bond length of O-O bond as Figure 2D shows. Due to the presence of the anti-bond orbital, O_2_ is activated and the energy required to open the O-O bond is thus reduced, which is helpful to the subsequent catalytic reaction. The PDOS also demonstrates that the 2π* orbital of O_2_ has a strong hybridization with the 3d-orbital of Fe, thus making the system more stable.

The value of the adsorption energy of the individual CO and O_2_ molecules on Fe@χ_3_-borophene are -2.44 eV and -2.21 eV, respectively. These large absolute values of the adsorption energy suggest that there are strong interactions between CO/O_2_ and Fe@χ3-borophene. The strong interaction between the gas molecules and borophene substrate is a prerequisite for the subsequent catalytic reaction of COOR. It should be noted that the difference between the adsorption energies of CO and O_2_ molecules is only 0.23 eV. As a result, either CO or O_2_ molecules can be absorbed firstly on the substrate. If a single CO is absorbed at the first time, the adsorption state of 2CO* will be formed when another CO approaches, as shown in Supplementary Figure S4A, where 2 C atoms are simultaneously bonded to Fe with the adsorption energy of -4.29 eV. We also simulate the structure of the classical co-adsorbed state of CO + O_2_* when a CO molecule is first absorbed and then an O_2_ molecule approaches (Supplementary Figure S4B). In this case, a Fe atom is bonded with two O atoms and a C atom at the same time with the adsorption energy of -3.15 eV. Since the adsorption energy of the CO + O_2_* is 1.14 eV smaller than the case of 2CO*, the CO + O_2_* is more difficult to occur. Another possible adsorption pathway is that a single O_2_ is first adsorbed on the substrate. In this case, an intermediate state of CO_3_* will easily form when CO approaches the system with a large adsorption energy of -5.24 eV, which is 2.09 eV more negative than the case of CO + O_2_*, so that the CO + O_2_* is unlikely to form in this case either. Furthermore, the adsorption energies of one or two CO_2_ molecules are -0.33 eV and -0.59 eV respectively, which is quite small compared to that of the other gas molecules. Only -0.02 e and 0.02 e are transferred between CO_2_ and the substrate, indicating that CO_2_ is physically adsorbed on the substrate by van der Waals interactions, and can be separated from the substrate very easily.

### Reaction mechanism of CO oxidation on Fe@χ_3_-borophene

According to the adsorption energy calculations of different gas molecules, the possible paths for COOR on the Fe@χ_3_-borophene are summed up as follows.

**E-R:**

$$O_{2}^{*}+CO\to CO_{3}^{*}$$

$$CO_{3}^{*}\to O^{*}+CO_{2}\;\mathrm{or C}O_{3}^{*}+CO\to 2CO_{2}$$

**TER:**

$$2CO^{*}+O_{2}\to OCO-OCO^{*}$$

$$OCO-OCO^{*}\to 2CO_{2}$$

where “*” represents the adsorption state.

1) E-R reaction

**FIGURE 3:**
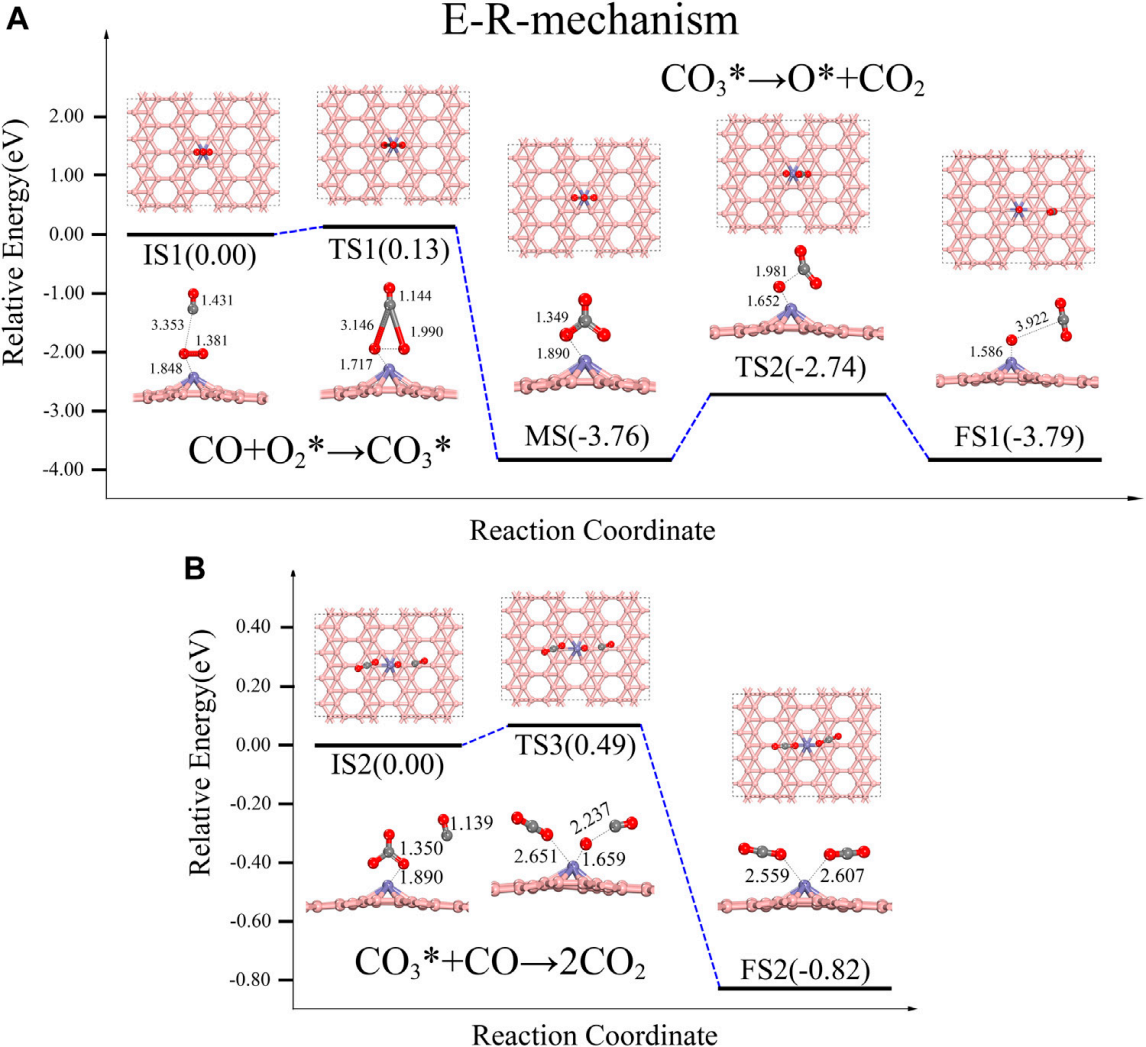
Energy distribution diagram under the E-R path and its corresponding structure diagram, the rate-determining step is IS2-FS2, turning over an energy barrier of 0.49 eV.

The E-R reaction path is shown in Figure 3. We place the CO molecule above the O_2_-Fe@χ_3_-borophene as the initial state (IS), representing the CO in the gas phase. In the first step of the reaction path, CO molecule combines with O_2_* to form an intermediate state (MS) CO_3_*. The energy barrier for this step is only 0.13 eV, and this reaction releases a large amount of heat (3.76 eV). In the conventional E-R reaction, the next step is the decomposition of CO_3_* into O* and CO_2_ (see Figure 3A). We simulate this reaction and find that the reaction barrier for CO_3_* to form CO_2_ by breaking the C-O bond directly requires 1.02 eV, which indicates that this step is unlikely to occur. However, there is another possible path for CO_3_* to decompose with lower energy barrier. Chen’s team found that the direct separation of CO_3_* requires 1.34 eV, while the path to form 2CO_2_ via another CO only needs to cross an energy barrier of 0.15 eV (Chen et al., 2020) when they studied the COOR on the 3Si-graphene-Ni. According to Chen’s result, here we also investigated the reaction path of forming 2CO_2_ by adding a second CO molecule in our system and the reaction pathway is shown in Figure 3B, where the reaction barrier is found to be reduced. In this reaction path way, the gas phase CO suspended around the CO_3_* is used as the IS. When the second CO molecule approaches, one of the C-O bonds in CO_3_* breaks. As a result, a CO_2_ is subsequently detached and the remaining single O atom combines with the CO molecule to form the final state (FS) 2CO_2_*. This reaction requires climbing an energy barrier of 0.49 eV and gives off 0.82 eV of heat as shown in Figure 3B, which is nearly half of the energy barrier of 1.02 eV for direct decomposition, indicating that the presence of gas-phase CO does effectively facilitate the dissociation of CO_3_*.

2) TER reaction

**FIGURE 4:**
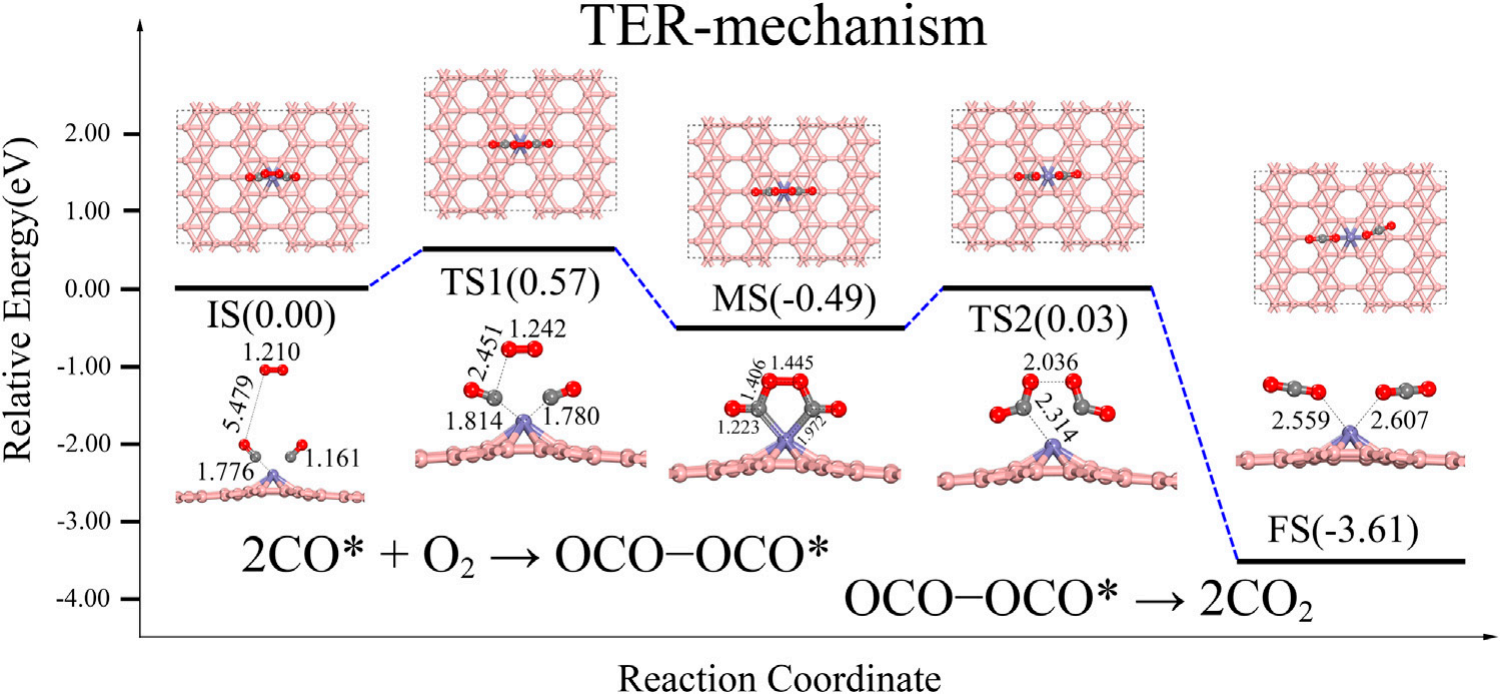
Energy distribution diagram under TER path and its corresponding structure diagram, the rate-determining step is IS-MS2 turning over an energy barrier of about 0.57 eV.

From the adsorption energies discussed above, it is apparent that two CO molecules are more likely to be adsorbed on Fe@χ_3_-borophene due to its ∼1.14 eV greater adsorption energy than that of the CO + O_2_*. Figure 4 shows that the TER reaction path, which starts from the IS of the reaction with the gas-phase O_2_ physiosorbed on 2CO-Fe@χ3-borophene. The two co-adsorbed CO effectively activate the gas-phase O_2_ to cross an energy barrier of about 0.57 eV. The product is an OCO-Fe-OCO* intermediate state releasing an energy of 0.49 eV. This OCO-Fe-OCO* intermediate state has a pentagonal structure, which is similar to the OCO-Pt-OCO* intermediate state reported by the Zhang team in the TER path on Pt/NG (Zhang et al., 2015). During the reaction, it is found that two oxygen atoms in O_2_ will approach the 2 C atoms of the CO molecules progressively and eventually form bonds to the C atom with a bond length of 1.406 Å.

The second step of the reaction path is that the intermediate state of OCO-Fe-OCO* proceeds to the final state of 2CO_2_*. It can be seen from Figure 4 that the MS needs to cross an energy barrier of about 0.52 eV to form two CO_2_, which is almost the same as the energy barrier of 0.57 eV required from IS to MS. The breaking of the O-O bond and C-Fe bond releases about 3.12 eV of heat. The O-O bond grows from 1.352 Å to 2.036 Å, and finally breaks completely, while the C-Fe bond grows from 1.973 Å to 2.314 Å, and finally breaks completely. The final product 2CO_2_ is physically adsorbed on the system and can be easily dissociated to allow the cycle to continue.

3) Discussion of catalytic results and electronic properties

Here we summarize the rate-determining steps (RDS) of both reactions described above. The important parameters of each reaction are listed in Table 2. In the E-R reaction, the RDS is2-FS2, climbing an energy barrier of about 0.49 eV, which is less than the energy barrier needed for the E-R reaction on Fe-SV-graphene (Li et al., 2010; Xu et al., 2020b). The RDS in the TER reaction is IS-MS, requiring an energy barrier of about 0.57 eV.

**TABLE 2:** The relevant parameters of each basic reaction path: the energy released by the reaction (E_reaction_), the energy barrier required for the reaction (E_barrier_), the charge transfer between reactants and substrates (Δq_1_), the charge obtained by χ_3_-borophene (Δq_2_), the charge loss of Fe atom (Δq_3_), the magnetic moment of the system (Moment), the distance between Fe atom and the χ_3_-borophene surface (d_Fe-bor_).

Reaction mechanisms	ER-step						TER-step				
	IS1	TS1	MS	IS2	TS3	FS2	IS	TS1	MS	TS2	FS
*E*_reaction_ (eV)		3.89			0.82			0.49		3.12	
*E*_barrier_ (eV)		0.13			0.49			0.57		0.52	
*Δq*_1_ (e)	−0.54	−0.84	−0.97	−0.97	−0.44	−0.02	−0.28	−0.39	−0.74	−0.68	−0.02
*Δq*_2_ (e)	−0.75	−0.60	−0.34	−0.32	−0.82	−1.36	−0.67	−0.65	−0.29	−0.54	−1.36
*Δq*_3_ (e)	1.29	1.44	1.31	1.29	1.23	1.38	0.95	1.04	1.03	1.22	1.38
*Moment* ( ${\text{μ}}_{\text{B}}$ )	2.0	1.8	0.0	0.0	0.7	3.0	2.0	1.8	0.0	2.1	3.0
*d*_Fe-bor_ (Å)	1.403	1.384	1.371	1.371	1.377	1.381	1.378	1.373	1.368	1.372	1.381

We analyze the electronic structural property of the system to provide deeper understanding for the reaction. As can be seen from the charge transfer between reactants and substrates (Δq_1_) in Table 2, the adsorbate will gain the largest number of electrons from the fe@χ_3_-borophene when O_2_ needs to be activated in both E-R and TER processes, with 0.97 e and 0.74 e obtained for the MS state in the E-R and TER reactions, respectively. Most of the obtained electrons will occupy the 2π* anti-bonding orbital of O_2_, leading to a longer bond length of O_2_, which makes subsequent catalytic reactions easier. In addition, it can be found from Δq_2_ which denotes the charge obtained by the χ_3_-borophene that it always gets electrons during the whole reaction process, and the number of electrons gained decreases significantly when the reaction requires a large number of electrons to activate O_2_, with 0.34 e and 0.29 e obtained for the MS state in the E-R and TER reactions, respectively. It is not difficult to find that χ_3_-borophene plays an important role in both donating and obtaining electrons in the whole reaction. Finally, it can be observed by the charge loss of Fe atom (Δq_3_) that the charge transfer of Fe atom is not very large in the E-R reaction. One possible reason is that Fe plays the role of a transport medium during the charge transfer process. Due to the strong electron deficiency of χ_3_-borophene, when a single Fe atom is adsorbed on the surface of χ_3_-borophene, the Fe atom will lose 1.34 e, and these electrons will be temporarily stored in the χ_3_-borophene. When O_2_ requires electrons to be activated in the subsequent catalytic process, charge transfer occurs from χ_3_-borophene to O_2_, so as to provide electrons to active the catalytic reaction. Therefore, not only does the Fe atom that directly interacts with the gas molecules play an important role in catalysis, but also the χ_3_-borophene as the substrate is the key to the catalytic reaction.

It is widely known that the analysis of the electronic state of the reaction path is of great significance for understanding the COOR process. It can be seen from Table 2 that no matter whether it is the E-R reaction or the TER reaction, the magnetic moments of the system undergo a process from presence to disappearance, and finally regain. O_2_ in the gas phase has a triplet state, resulting in a total magnetic moment of 2.0 ${\text{μ}}_{\text{B}}$ when O_2_ is initially adsorbed. As the reaction processes, charge transfer occurs between the adsorbate, Fe atom and the χ_3_-borophene, to promote the annihilation of the magnetic moment. Finally, the formation of CO_2_ brings the overall magnetic moment back to the original 3.0 ${\text{μ}}_{\text{B}}$ of Fe@χ_3_-borophene.

During the COOR catalytic reaction, the distance between the Fe atom and the χ_3_-borophene surface is slightly changed. The initial distance is 1.381 Å after the geometry optimization. In the catalytic reaction, the distance of the Fe atom from the χ_3_-borophene surface varies as shown in Table 2. The distances show a pattern of first getting smaller and then larger.

### The influence of temperature

**FIGURE 5:**
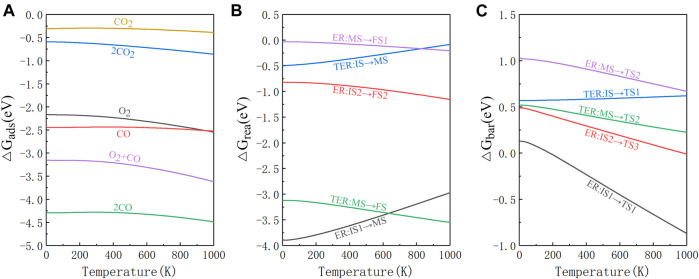
The change in Gibbs free energy of each reaction path at 0–1000 K. **(A)** The gas molecules adsorption energy. **(B)** The energy released by the reaction. **(C)** The energy barrier required for the reaction.

In order to get a more realistic picture of the catalytic reaction, it is important to consider the effect of temperature. According to Peng team’s research, χ_3_-borophene remains thermodynamically stable at 1000 K (Peng et al., 2017), thus we plot the change of adsorption energy, reaction energy, and energy barrier for the whole system at 0–1000 K as shown in Figure 5. As can be seen from Figure 5A, when the temperature ranges from 0 to 300 K, the value of adsorption energy is almost the same compared to the adsorption energy at 0 K. When the temperature is greater than 300 K, the absolute value of the adsorption energy increases, but the order of the adsorption energy values does not change. The absolute value of the adsorption energy of O_2_+CO* is still about 1 eV lower than 2CO*, so the L-H reaction will not occur, indicating that the reaction path we obtained at 0 K is unchanged. The changes in the Gibbs free energy of the E-R and TER reactions are all negative, so all steps are exothermic as depicted in Figure 5B. Finally in Figure 5C we find that as the temperature reaches 200 K, the reaction energy barrier of IS1→TS1 in the E-R reaction becomes negative. It indicates that CO_3_* is more likely to form spontaneously under the influence of temperature. The energy barrier of MS→TS2 is always greater than IS2→TS3 in the E-R reaction, so the E-R reaction still requires a second external CO to participate in the presence of temperature. The energy barrier of IS→TS1 in the TER reaction increases slightly with temperature, but the value of the energy barrier is not large, and the reaction can still proceed. Overall, the RDS of each reaction step does not change with the presence of temperature and most of the reaction energy barriers decrease significantly with the increasing temperature.

### Reaction rate

Table 3 shows the specific time required for each step of the reaction process obtained by the Arrhenius law. In the E-R reaction, it can be seen that the time required for IS1-MS is very short at any temperature, which is consistent with the low energy barrier. In addition, the time required for IS2-FS2 is much less than that for MS-FS1, which means that the direct dissociation of CO_3_* is very difficult and the second CO is always required in the actual reaction. In the TER reaction, when the temperature is 200 K, the time taken by IS-MS is about 3.11 × 10^2^ s, thus the reaction is not easy to occur. However, the entire TER reaction can proceed normally at higher temperature. In summary, the reaction rate of the E-R reaction is fast at any temperature, while the TER reaction is more suitable when temperature is above 200 K.

**TABLE 3:** Time required for each step of the reaction path in E-R and TER reaction at 200 K, 298.15 K, 400 K, 500 K.

Reaction step Reaction time	E-R-step			TER-step	
	IS1-MS	MS-FS1	IS2-FS2	IS-MS	MS-FS
τ (s)/200 K	3.42 × 10^−13^	5.64 × 10^12^	9.88 × 10^−1^	3.11 × 10^2^	9.88 × 10^−1^
τ (s)/298.15 K	8.72 × 10^−15^	1.04 × 10^4^	3.14 × 10^−5^	6.04 × 10^−3^	3.14 × 10^−5^
τ (s)/400 K	1.17 × 10^−15^	2.97 × 10^−1^	1.47 × 10^−7^	2.30 × 10^−5^	1.47 × 10^−7^
τ (s)/500 K	3.59 × 10^−16^	6.07 × 10^−4^	6.34 × 10^−9^	8.78 × 10^−7^	6.34 × 10^−9^

## Conclusion

The DFT calculations have been performed to investigate the mechanism of CO oxidation on Fe@χ_3_-borophene. In this work we have ensured the stability of the system with formation energy calculation, diffusion path calculation and *Ab-Initio* molecular dynamics simulation. Two possible reaction paths have been identified by absorption energy comparisons and their energy barriers have been calculated. The results show that the COOR on the Fe@χ_3_-borophene substrate has good catalytic performance. Further electronic structure analysis indicates that charge transfers between χ_3_-borophene and CO, O_2_ and CO_2_ molecules are the major cause of both the low energy barrier and magnetic moment difference. First-principles study of Fe@χ_3_-borophene as a catalyst for COOR provides new possibilities and ideas for the COOR, and proposes new opportunities for the application of borophene in the field of heterogeneous catalysis.

## Data Availability

The original contributions presented in the study are included in the article/Supplementary Material, further inquiries can be directed to the corresponding authors.
