# Supplementary material for: Fe@χ3-borophene as a promising catalyst for CO oxidation reaction: A first-principles study
Source: Front Chem. 2022 Sep 13;10:1008332. doi: 10.3389/fchem.2022.1008332 (PMC9513182; doi:10.3389/fchem.2022.1008332)
Supplement: Supplementary file 1 [file DataSheet1.docx]

Supporting Information for

# Fe@χ_3_-borophene as a Catalyst for CO Oxidation Reaction: A First-principles Study

Jianwei Han^1^, Weiyue Bian^1^, Yue-Yu Zhang^1, 2,^ *, Meng Zhang^1,^ *

**Affiliations:**

^1^School of Physics, East China University of Science and Technology, Shanghai 200237, China

^2^Wenzhou Institute, University of Chinese Academy of Sciences, Wenzhou, Zhejiang 325000, China; Oujiang Laboratory (Zhejiang Lab for Regenerative Medicine, Vision and Brain Health), Wenzhou, Zhejiang 325001, China

In the supplementary section we first showed the initial and final adsorption sites of Fe atom absorbed on χ_3_-borophene, then we draw the specific images and energies of nFe(n=1-4) and Fe_n_(n=1-4) clusters adsorbed on the graphene and χ_3_-borophene, respectively. After that, we gave the lowest energy adsorption positions of the different gas molecules including 2CO, CO+O_2_, CO_3_, CO_2_ and 2CO_2_ adsorbed on Fe@χ_3_-borophene.


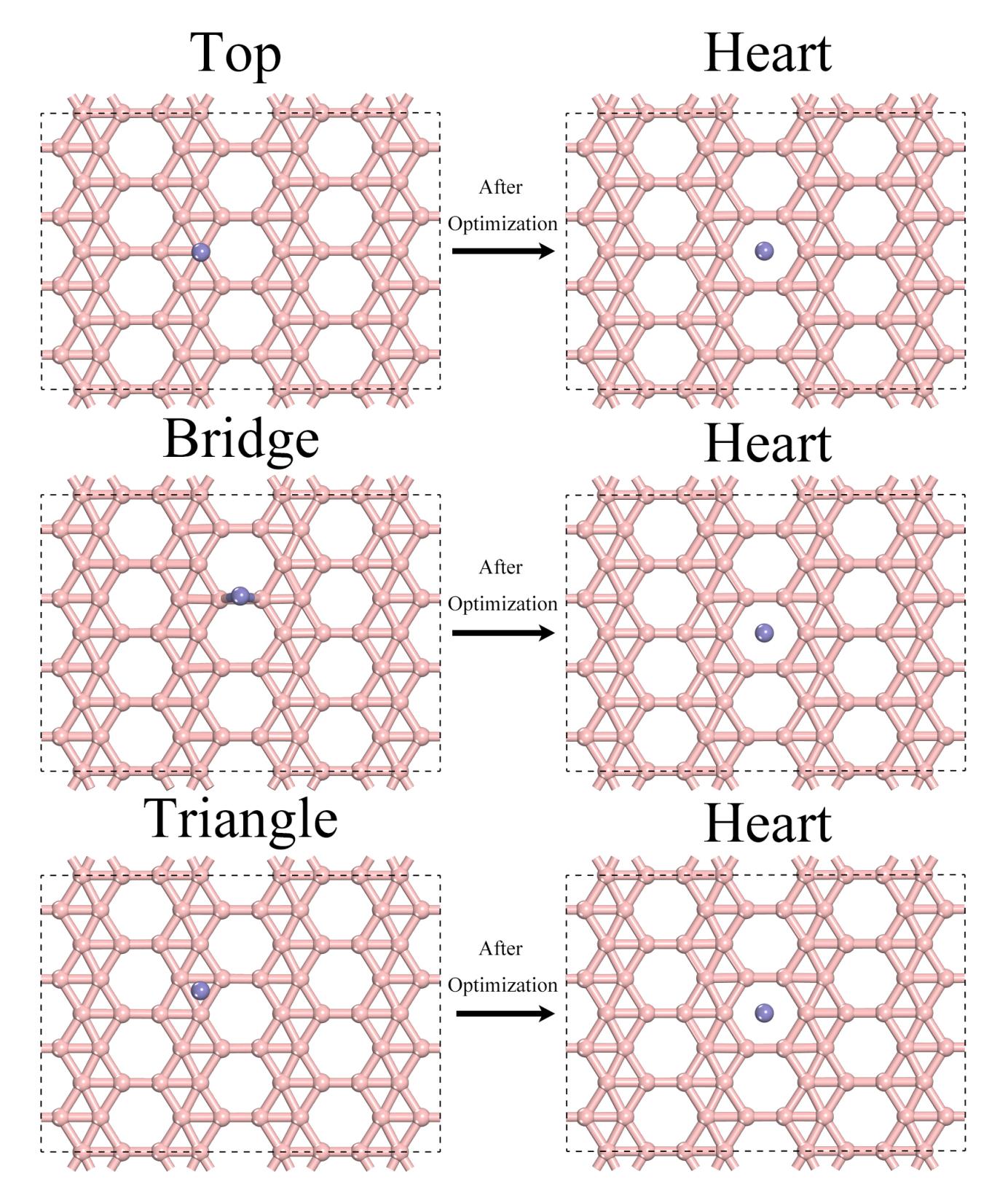


Figure S1 Top views of the most stable configurations of single Fe atom adsorbed on χ_3_-borophene with the test adsorption site on the left and the final adsorption site on the right


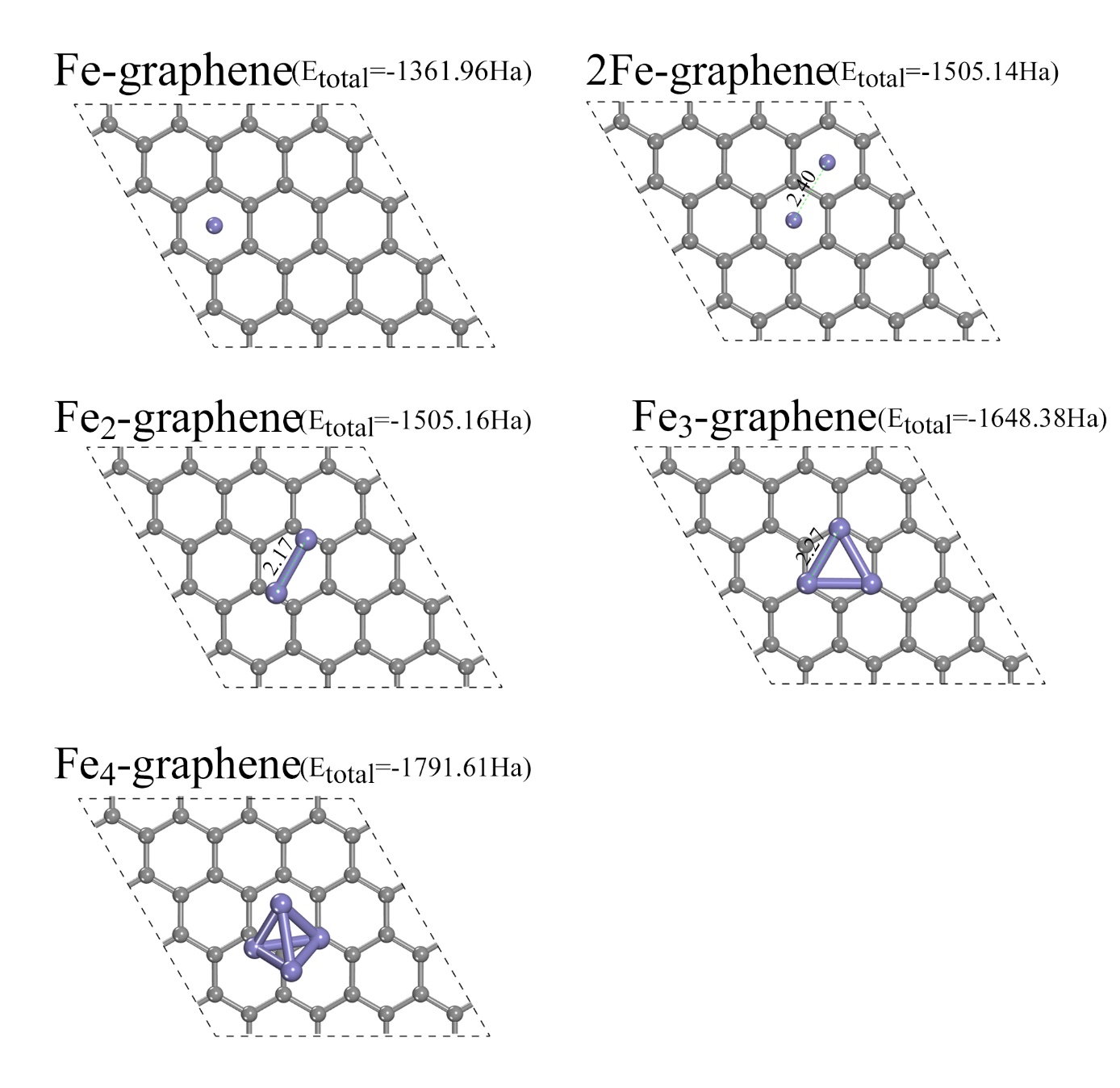


Figure S2 Structural diagrams and the total energy of nFe (n=1,2) and Fe_n_ (n=2-4) clusters adsorbed on graphene where Fe atoms form clusters spontaneously in the case of nFe (n=3,4)


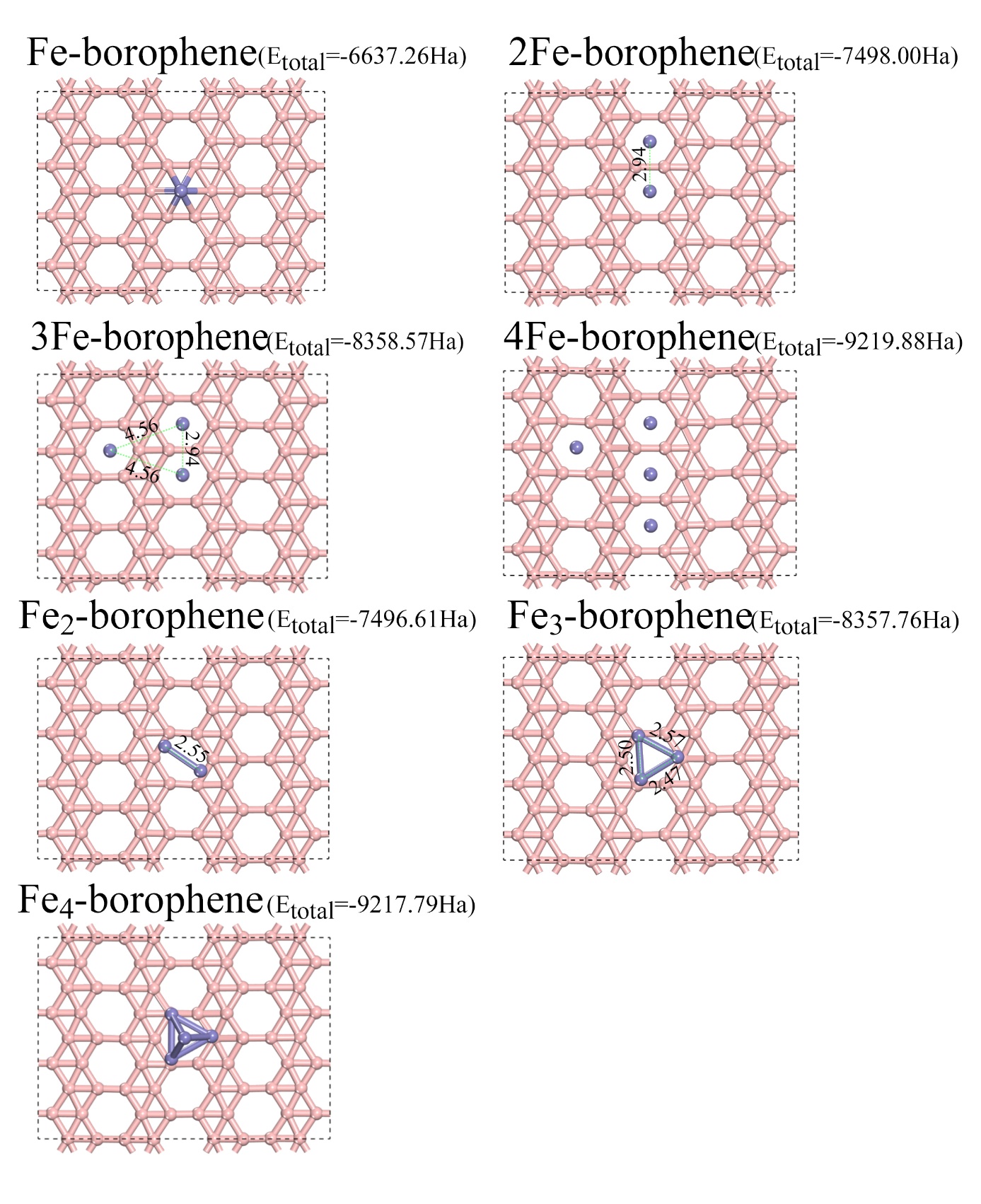


Figure S3 Structural diagrams and the total energy of nFe (n=1-4) and Fe_n_ (n=2-4) adsorbed on χ_3_-borophene


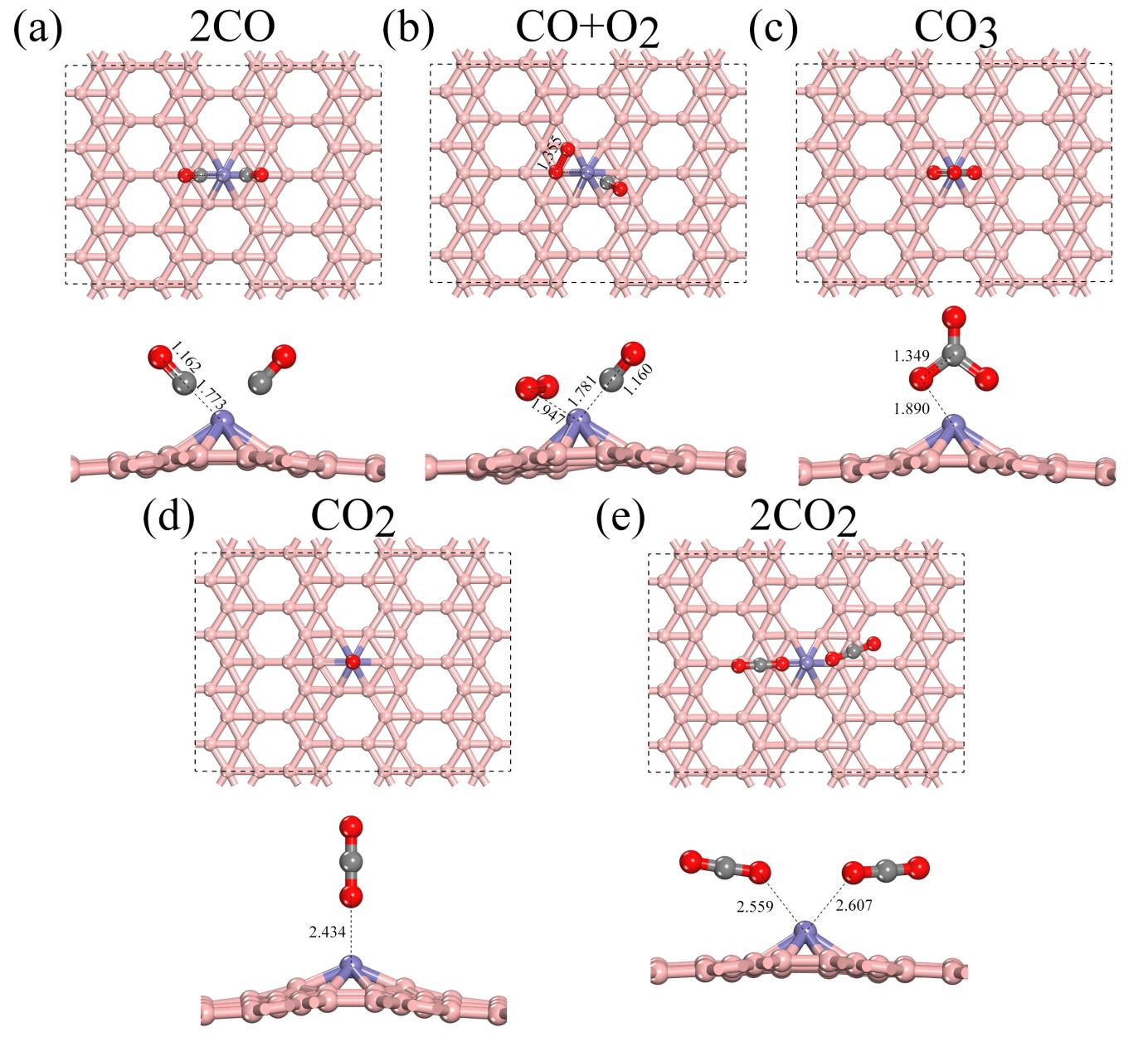


Figure S4 Structure diagrams of different gas molecules adsorbed on Fe@χ_3_-borophene
